# Colon or semicolon: gut sampling microdevices for omics insights

**DOI:** 10.1038/s41522-024-00536-2

**Published:** 2024-10-02

**Authors:** Sunil Nagpal, Sarvesh Kumar Srivastava

**Affiliations:** 1 TCS Research, Tata Consultancy Services Ltd, Pune, India; 2 CSIR-Institute of Genomics and Integrative Biology (CSIR-IGIB), New Delhi, India; 3 Centre for Biomedical Engineering, Indian Institute of Technology Delhi, New Delhi, India; 4 Department of Biomedical Engineering, All India Institute of Medical Sciences, New Delhi, India

**Keywords:** Microbiota, Next-generation sequencing

## Abstract

Ingestible microdevices represent a breakthrough in non-invasive sampling of the human gastrointestinal (GI) tract. By capturing the native spatiotemporal microbiome and intricate biochemical gradients, these devices allow a non-invasive multi-omic access to the unperturbed host-microbiota crosstalk, immune/nutritional landscapes and gut-organ connections. We present the current progress of GI sampling microdevices towards *personalized metabolism* and fostering collaboration among clinicians, engineers, and data scientists.

## Introduction

**Fig. 1:**
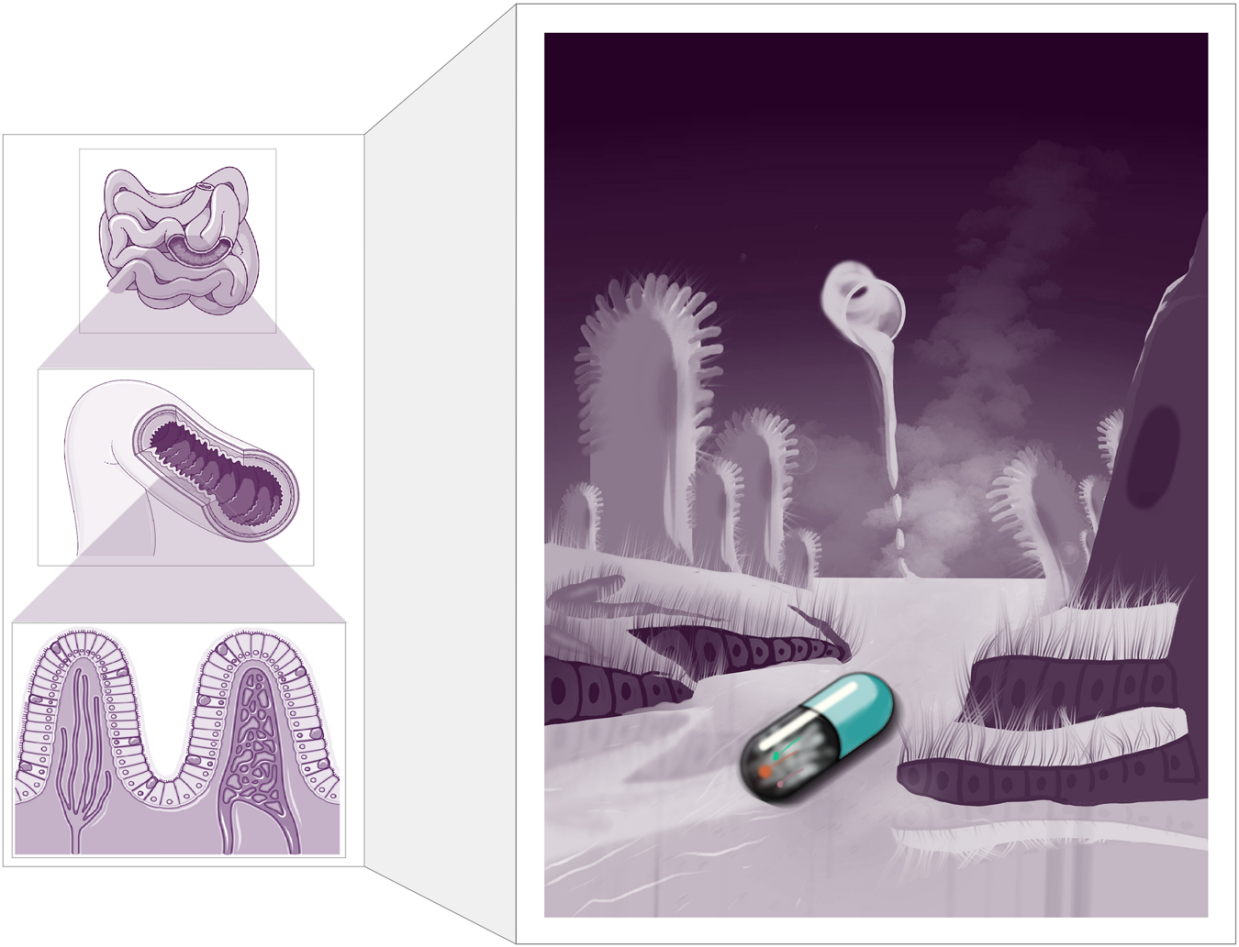
Graphical illustration of the complex topographical and biochemical microenvironment of human gastrointestinal (GI) tract, represented by zoomed in panels of luminal biogeography (left) and an artistic impression of this unique niche (right). The artistic impression also introduces the element of a pill/capsule entrapping the surrounding environment, which represents the overarching theme of this perspective. A portion of the illustration (left panel) is adapted from Servier Medical Art by Servier and licensed under a Creative Commons Attribution 3.0 Unported License. The remaining panel (right) has been drawn by the co-author (SN) using Procreate and Inkscape.

An alien planet with a unique ridge-like topography, filled with gooey viscous fluids, streams of acids, and an unknown abundance of life forms interacting together. This is not a sci-fi movie plot but the gastrointestinal (GI) tract’s scientific reality^1^, as depicted in Fig. 1. The 32–40 m^2^ surface of the ~10 m-long tube-like structure of the human GI tract is a living microcosmos of its own^2,3^. Shaped by the biochemical gradients of nutrients, pH, oxygen, digestive enzymes, salts, minerals, and more, the “life-supporting” terrain of the GI tract offers excellent habitat for a large variety of microorganisms^3–5^. From food digestion to immune system regulation and protection against pathogens, the human gut microbiome, constituted by trillions of microbial cells, far exceeds the encoded genetic content and biochemical transformational capabilities of humans^5,6^. Despite significant progress in next-generation sequencing, a bottleneck remains in the non-invasive isolation of the residing GI microbiome and the required biochemical techniques for sample processing (in microlitre volumes). In this context, the in-situ gut sampling microdevices represent a significant breakthrough. These microbotic devices address critical challenges such as cross-contamination and low spatial resolution, commonly associated with conventional gut sampling techniques like endoscopy, ileostomy, or fecal sampling^7^. This perspective discusses the current state-of-the-art in non-invasive gut sampling microdevices^8^ towards spatiotemporal sampling of the GI tract, emphasizing the effects of regional intestinal physiology, and related data trends emerging from the subsequent multi-omic analyses. We advocate for comprehensive policy discussions on the implementation of GI traversing microdevices and the ethical considerations surrounding human microbiome data.

**Fig. 2:**
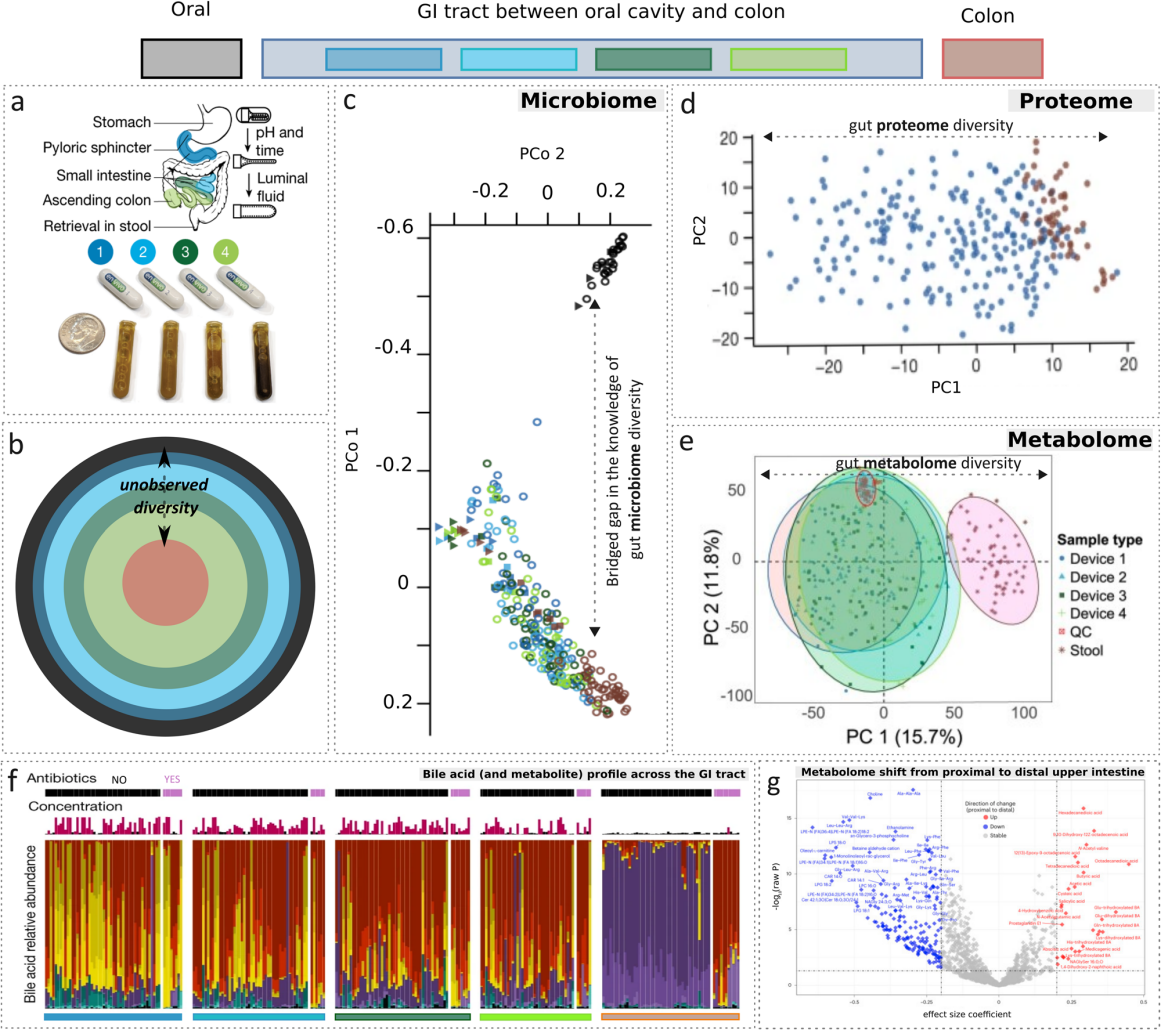
Gut sampling microdevices fill the gaps in the knowledge of taxonomic and functional microbial diversity of GI tract. **a** Ingestible capsule-sized gut sampling microdevices with a schematic representation of the intended sampling locations (top), device design (middle) pre-ingestion, and respective post-sample retrieval state (bottom). The US dime in the figure highlights the scale of the CapScan microdevice. Shared under a Creative Commons Attribution 4.0 International License with copyrights reserved with the authors^9^. **b** Schematic of the idea that gut microbiome diversity is not fully resolved when targeting only the colon (through ex-situ stool samples) or only the oral cavity (through ex-situ saliva samples). Gaps in knowledge remain given the unobserved diversity of other microniches of the GI tract. Colors map to the locations in the gut, highlighted in 2a and in the legend on the top of the figure. Size of the circles map to the proximity to oral cavity (indicator of the depth of the GI tract). Notably, the circles may also be viewed from the perspective of radial diversity, with the size representing proximity to the vasculature and hence oxygen availability, while inner circle being proximal to the lumen with lower oxygen availability. The longitudinal and radial microbial/multi-omic diversity remains to be bridged. **c** Beta-diversity (using Canberra distance for PCoA) of the samples collected by the four microdevices for the observed taxonomic profiles, as compared to that observed in ex-situ counterparts (stool and saliva samples). The four devices capture a gradient of unobserved microbial diversity across different regions of the gut and at different time points of sample collection. Shared under a Creative Commons Attribution 4.0 International License with copyrights reserved with the authors^9^. **d** Beta-diversity (using PCA) of the device collected gut samples and ex-situ stool samples, with respect to the normalized human protein abundances (proteome), highlighting the gaps filled by the microdevices in capturing the gradients of functional diversity as well. Adapted from ref. ^9^ with permission. **e** PCA of the metabolome profiled for the same set of stool and device collected samples, additionally highlighting the previously unobserved metabolic diversity along the length of GI tract. Adapted from extended data of^12^ with permission. **f** Stack bar plot representing the variations in proportions of various bile acids observed in the samples collected by the devices as well as through ex-situ stool sampling (color coded horizontal bars under the plot indicate sampling location). Colors of the bars of the stack represent various types of bile acids. Brown-yellow color palette pertains to primary bile acids and magenta-turquoise palette pertains to secondary bile acids. Set of subjects exposed to antibiotics within six months prior to sampling are indicated by YES/NO. Shared under a Creative Commons Attribution 4.0 International License with copyrights reserved with the authors^9^ **g** Volcano plot depicting the differential as well as overlapping metabolome profile between proximal and distal regions of the gut (excluding colon) obtained using linear mixed-effect modeling (LMM). For example device 1 and device 4 in Fig. 2a would pertain to proximal and distal regions respectively. A significance threshold of *P* < 0.05 (testing 1,182 metabolites), indicated by horizontal dashed line, was employed. Red, blue and gray colored points indicate upregulated, downregulated and stable metabolites while transitioning from proximal to distal gut. Shared under a Creative Commons Attribution 4.0 International License with copyrights reserved with the authors^12^. (For interpretation of the references to color in this figure legend, the reader is referred to the web version of this article.).

Recently, Shalon et al. demonstrated an orally ingestible microdevice (CapScan®) for sampling the human intestinal environment under physiological conditions^9^. The device includes a collapsed bladder with a one-way valve within a dissolvable capsule with a pH-sensitive enteric coating. This coating safeguards the bladder from expanding in the stomach until it reaches the small intestine, where the shift in the acid gradient triggers the disintegration of the enteric coating. At the designated pH level, regulated by the enteric coat thickness and polymer composition, the valve draw in luminal fluids (400 µl) thereby expanding the collection bladder. By targeting the actuation of multiple devices along the length of the GI tract (Fig. 2a), the study demonstrates that concomitantly collected fecal samples provide a relatively localized and limited snapshot of the gut microenvironment (Fig. 2b–e). This is confirmed in the gradient of microbial beta-diversity (i.e., inter-sample variations in microbial composition) of the samples collected by the microdevices transiting different GI sites at different time points of digestion (Fig. 2c). The closely clustered oral and fecal samples, despite longitudinal sampling, on the other hand, indicated the limited accuracy of ex-situ samples in capturing the native spectrum of spatiotemporal variations in the gut microbiota. This ability of the in-situ site-directed samples to bridge “the unobserved diversity” or gaps in the current knowledge (schematically represented by different colors for different biogeographical regions of the gut in Fig. 2b), instead extends beyond taxonomy to the gut proteome (Fig. 2d) as well as the metabolome (Fig. 2e). The latter are important, given that gut bacterial diversity contributes to the diverse proteomic and (secondary) metabolic pools in the GI tract. Production of secondary bile acids, which exhibit a heightened affinity for the activation of various host nuclear receptors compared to the primary bile acids, is a pertinent example^10^. These bile acid nuclear receptors extend beyond the enterohepatic circulation to tissues like the heart, adipose tissue, and potentially the kidney, exerting a systemic impact on the host’s health. Shalon and colleagues, for example, identified novel trends in the gut virome, host-proteome, and metabolome, especially of microbially conjugated bile acids, which remained hitherto unobserved in the matched ex-situ samples (Fig. 2f). This supports a renewed focus on microbial conjugation of the bile acids in the intestine, now known to significantly impact bile acid signaling in the liver and potentially other tissues, thereby shaping the pathophysiology^11^.

In a companion pilot human study (*n* = 15) with the same CapScan® device, Folz et al. probed the spatiotemporal variation of the upper intestinal luminal metabolome during digestion. They identified 1909 metabolites, including sulfonolipids and fatty acid esters of hydroxy fatty acids (FAHFA)^12^. Furthermore, it was observed that the stool and intestinal metabolomes, apart from a stable overlapping baseline, differ dramatically (Fig. 2g)—potentially linking the variation captured by the devices (Fig. 2a, e) to the differences in the diet as well as the host and microbial metabolism across the upper and lower GI tract. Notably, site-specific peculiarities in bile acid metabolites (Fig. 2f), the levels of bioactive FAHFAs, sulfonolipids, and other microbially related metabolites were additionally apparent across the entire GI tract in the subjects who had consumed antibiotics within six months before sampling. Figure 1f for example depicts the antibiotic exposure linked perturbations in microbial metabolism of bile acids. The levels of primary bile acids (shown in brown-yellow bars) were observed to be elevated, with a drop in secondary bile acid levels (magenta-turquoise) in subjects with antibiotic treatment as compared to those without prior antibiotic exposure. It may be noted that dietary components can also impact the bile acid pool similar to antibiotics due to their effects on bile salt metabolizing members of the microbiome^13^. The diet and microbiota-shaped bile acids have both direct antimicrobial effects on gut microbes^14^ and indirect effects through Farnesoid X receptor-induced antimicrobial peptides^15^.

From a biomedical device’s perspective, these observations offer a dual strategy to unveil multi-omic diversity and the differential impact of interventions, like antibiotics, across the gut anatomy in assessing the microbial diversity. Essentially—colon, or beyond it, i.e., semi-colon—is the fundamental question currently driving the non-invasive gut sampling research.

Nevertheless, non-invasive in-situ sampling of the GI tract comes with its own set of challenges. Broadly, these challenges are of both design and biochemical analyses. While the former entails the constraints on size, actuation mechanisms, sample isolation, and sampling volume for the devices, the latter pertains to the need to keep the native biology of the sampled microliter volumes intact. All of this is within an implied understanding of device safety i.e., no accidental retention or perforation of the GI mucosa. The timeline of progress in developing translatable gut-sampling devices (Table 1) is much driven by these challenges^16–25^.

**Table 1:** Advancements in the design and development of gut sampling microdevices in the last 30 years

SNo.	(Brand) Name	Mechanism	Targeted site	Year [Reference]
1	Intestinal fluid sampler	Vacuum suction-based oral pill with a dissolvable seal of epoxy compounds for sampling initiation	Luminal content of desired site of GI tract based on selection of the epoxy	1996,^25^
2	In-vivo sampler	A vaccum and chemical seal based multi-copartment microchannel device for fluid collection throughout GI tract	Luminal content of multiple sites in a single collection event along the length of GI tract	2006,^23^
3	MEMS device	Remote controlled microcapsule, with mechanic movement of a piston in a microelectromechanical system (MEMS)	Luminal content of required site of small intestine	2008,^24^
4	Internal substance sampling device	A flexi-control (both passive and active actuation) microcapsule system with spring based expandable wall for large volume (~1.5 ml) fluid collection	Luminal content of required site of small intestine	2014,^22^
5	IntelliCap®	Enteric-coated, pH guided smart capsule system armed with sensors for physiological parameters and a quencher in the piston pump based collection container for sample preservation	Luminal content of required site of GI tract (originally designed for targeted drug delivery)	2014,^21^
6	Osmotic pill	Enteric-coated 3D-printed pill containing a salt chamber separated from helical collection channels by a semipermeable membrane for osmotic movement of intestinal fluid	Luminal content of desired site of GI tract	2019,^20^
7	TRAP Microbots	Self-propelled micromotor system with self-polymerizing hydrogel-forming reaction system for gut microenvironment entrapment and preservation	Luminal content of the desired GI site through propulsion system manipulation	2019,^19^
8	Smart capsule	A hydrogel-based 3D printed microscapsule with flexible membrane, that captures the luminal sample in the swelling polymeric matrix	Luminal content of any site of GI tract	2020,^18^
9	Untethered robotic capsule	Remote-controlled robotic device with spring actuators of shape memory alloy (SMA) to function against peristaltic forces	GI tract mucosa	2021,^17^
10	CapScan	Enteric-coated pH regulated microcapsule with a collapsed bladder and a one-way valve	Luminal content of the desired site of GI tract	2023,^9^

While the initial, vacuum-based, passive sampling designs suffered from a foundational limitation of multi-site sample mixing after disintegration/dissolution of epoxy-coated inlet-chamber seals^25^, re-sealing valves and osmotic isolation of fluids in subsequently evolved devices ensured that the fluids were not exposed post-collection^20,23^. Recent advancements have instead led to remote-controlled active sampling devices, adding marked precision to the site and time (including cessation) of sample collection within the gut milieu^18,24^.

Firstly, to ensure patient compliance, these devices are designed to be non-invasive and scaled/contoured to mimic conventional pills/capsules (Size 0-00). This invariably translates into small sampling volumes (200–400 µl)^9,20^. Given the small volumes and the residence time between device actuation and device harvesting under elevated body temperatures, the challenge is to halt further metabolism of collected digesta inside the device. The latter is vital for sample integrity, ensuring attaining the all-important representative/native state of the sampled microbiome (composition). In fact, one of the critical challenges when microsampling the lower GI tract is that the degradation of the dietary components continues as the device traverses downstream despite being trapped inside a device – much like a miniaturized incubator. In this regard, Rios-Morales et al. investigated a buffer cocktail for metabolic quenching and analyzed the quenched intestinal samples from the gastrointestinal sampling capsules^26^. The reagent was based on a bacterial lysis buffer with the addition of several components to denature enzymes. Overall, the quenching reagent effectively blocked fructooligosaccharide and galactooligosaccharide degradation in ileostomy samples at 1:5 v/v. Also, this preserved 16S rRNA copy number and microbial composition. The authors claimed that the *quenching cocktail* comprising NaCl, EDTA, Tris, SDS, and urea (at pH 8.5) could be preloaded in gastrointestinal capsules to block further fermentation (degradation) of fibres in the sample microvolumes. This is very relevant given dietary fibers, like pectin, have a direct effect on the GI immune barrier^27^.

**Fig. 3:**
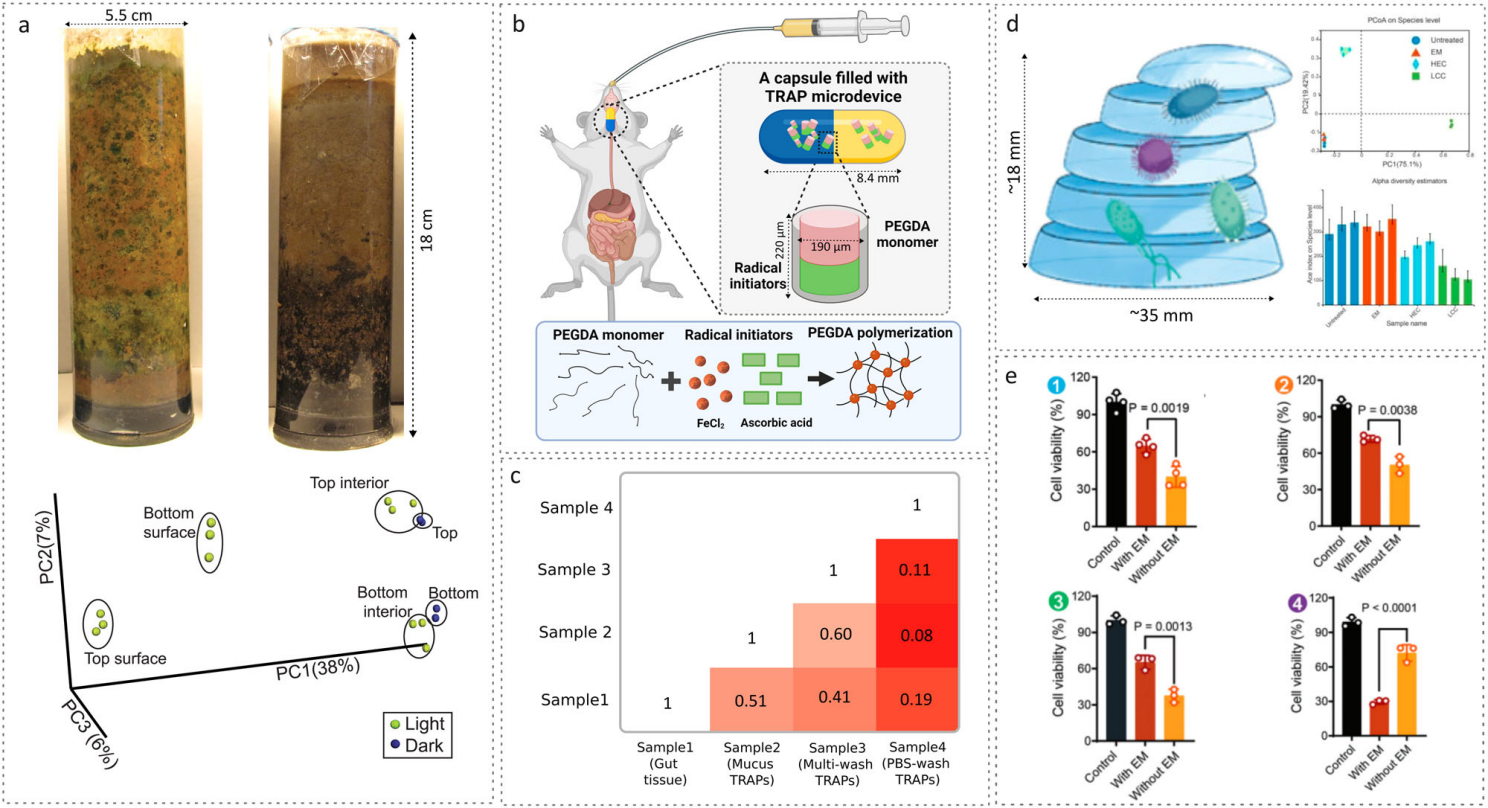
Native environment entrapment for ex-situ culturing of the innate microbiome. **a** Macroscopic Winogradsky columns (18 cm × 5.5 cm) with stacked layers of soil from the natural environment (top), each layer sustaining the, hitherto unculturable, native microbiome specific to the niches in each stratum as depicted by the spatially segregated clusters in beta-diversity plot for layer-specific samples (bottom). Shared under a Creative Commons Attribution License with copyrights reserved with the authors^32^. **b** Schematic diagram of microscopic (220 µm × 190 µm) TRAP microdevice administered orally in enteric-coated 8.4 mm long gelatin capsule. Reprinted (adapted) with permission from ref. ^16^ and figure created with BioRender.com. Copyright 2020 American Chemical Society. Each device can undergo self-polymerization upon contact with luminal fluids to form a container of PEGDA hydrogel that can trap the environment in vicinity. **3c)** Bray−Curtis dissimilarity matrix derived from microbiota profiles of TRAP retrieved samples from gut tissue, gut mucosa and washed TRAP devices, indicating the ability to capture and compare anatomically distinct but closely related microenvironments of the GI tract. Reprinted (adapted) with permission from ref. ^16^. **d** Schematic representation of the millimeter scale (35 mm x 18 mm) ex-situ multi-layer gradient hydrogel, where microbiota-inoculated layers mimic the physiological gradients of the GI tract (left). Shared with permission from^34^ John Wiley & Sons. Beta-diversity. Top right) and alpha diversity (bottom right) analysis determining the effect of EM (gradient hydrogel-based engineered microbiota), LCC (liquid culture community), and HEC (hydrogel encapsulated community) in maintaining the native gut microbial community (untreated). Reprinted with permission from the supplementary content of ref. ^34^. **e** Bar-plots indicating the effectiveness of anti-cancer mitoxantrone (1), ganetespib (2), and CI1040 (3) and CB1954 (4) in affecting tumor cell viability with and without gradient hydrogel-based engineered microbiota (EM) incubation. Reprinted from ref. ^34^ with permission from John Wiley & Sons.

The second area must include culturing the isolated microbiome under laboratory conditions. The common misconception that most (human as well as environmental) microbiomes are not culturable needs an alternate perspective^28–30^. Winogradsky columns invented in the 1800s by Sergei Winogradsky^31^ in fact proved the latter more than two centuries ago by culturing a large diversity of hitherto unculturable microorganisms in a (typically 0.5–5 feet) long column of glass (Fig. 3a)^32^ with strata of sediments obtained from the natural ecosystem—the native environment of the said microbes across different layers of the soil (Fig. 3a, PCoA)^32^. The inability to culture a majority of the microbiota (including gut microbiome) could stems from the limited understanding of the diverse nutritional requirements of this abundant microflora^29^. In fact, human GI tract, unlike environmental niches like soil, characterized by the topographical complexities alone, is a very challenging environment to even attempt a replication of a Winogradsky-like ex-situ set-up. In this regard, Thread-Like Radical-Polymerization via Autonomously Propelled (TRAP) technology employs micrometer-sized cylinders (Fig. 3b), packed with a hydrogel-forming pre-polymer mix that isolates the microbiome and, to an extent, its microenvironment via a hydrogel-forming self-polymerization reaction^16,19^. A post-hoc metagenomic sequencing of the gut-tissue and mucosa microenvironment retrieved by TRAPs (in Sprague−Dawley rats) revealed the ability to capture the differences (and relatedness) of the said anatomically close but complex regions of the gut (Fig. 3c). Sustenance and identification of *Romboutsia sp*., native to the intestinal crypts buried deep into the mucus, inside the mucosa TRAPs was an exciting finding towards deep microbiota profiling of the gut^16^. Comprehensive multi-omic studies on such entrapped samples may further validate the suitability of this technology in ex-situ preservation of the complex innate intestinal microenvironment. Importantly, given these soft polymeric gels are re-culturable, TRAP-like technologies promise to open new alternatives for accelerating human microbiota culturing, enabling mechanistic studies, verifying genomic predictions, and translating them as novel therapeutics^28^.

Thirdly, in areas where the entrapment of the natural environment and ex-situ maintenance of gut microbiota hold immense significance, is drug-microbiome cross talk. Peppercorn and Goldman, in 1972 demonstrated the role of intestinal bacteria in metabolizing the anti-inflammatory drug salicylazosulfapyridine^33^. Since then, the impact of various drugs on the native microbiota has been a high priority in human health and pharmaceutical research. This includes unexpected outcomes (including side- effects) and the reciprocal metabolic role of microbes in influencing drug efficacy. However, in-vivo microbiota modulation and continuous monitoring encounter obstacles given gut microbiota and drug molecules interact in difficult-to-reach GI microniches^34^. To overcome the challenge, building on the hydrogel-based microbiota encapsulation setup, Zhang et al. have demonstrated a 3D experimental model for bridging microbiota-drug interaction research^34^. They developed a multi-layered gradient hydrogel using spin coating and photo-initiated polymerization (Fig. 3d). This hydrogel was claimed to mimic and maintain the physiological, immunological, and microbial gradients of the gut microenvironment by incorporating mucin, intestinal bacteria, and EGFP-HEK293 cells. Designed through an intricate layer-by-layer arrangement of mucin-poly(sodium 4-styrenesulfonate (PSS)), poly-(ethylene glycol) diacrylate (PEGDA)-intestinal bacteria and polyallylamine hydrochloride (PAH)-PEGDA, the opposite charges of PAH and PSS were claimed to stabilize the gradient of layers, such that mucin-supported bacterial growth, while PAH prevented bacterial crossover at the interface. A close conformation of the native microbial (beta and alpha) diversity of the gut with that observed in this engineered microbiota or EM (Fig. 3d, insets) validated the gut-mimicking capability of the designed hydrogel model. The authors employed this validated (EM) model to assess the cytotoxic impact of chemotherapeutic drugs on tumor cells both with and without the EM. Upon EM incubation, the effectiveness of anti-cancer mitoxantrone, ganetespib, and CI1040 diminished by 42%, 30%, and 43%, respectively, while CB1954’s efficacy increased by 60% (Fig. 3e subplot 1–4, respectively). Finally, the authors also demonstrated dosage form modification by incorporating improved therapeutic effects in orthotopic tumor models with the patient-derived gut microbiota.

The engineering of an ex-situ hydrogel model (engineered microbiota or EM) that closely mimics the in-situ conditions of human gut, clearly opens avenues for not only the non-invasive interventional studies but also expanding the microbiota culturing capabilities, for highly optimized clinical outcomes. This is also vital for standardizing sequencing and even studies aiming to develop in-silico models of gut through three notable advantages: (i) helping overcome biases due to “microdevice contamination”, “sample modification” (as it traverses down the GI tract) and “storage/recovery” of the isolated luminal fluids (ii) providing ex-situ access to the in-situ microenvironment, enabling convenient interventional studies (iii) generating reliable native state multi-omic data required for physiological simulations and developing in-silico models.

The fourth area related to gut sampling microdevices, though currently underutilized, involves the vast amounts of biochemical, (meta)genomic, (meta)transcriptomic, proteomic, and metabolomic data, and the emerging role of artificial intelligence in multi-omic data analysis. Integrating these minimally perturbed, simultaneously captured datasets to train intelligent machines can enable the development of reliable multi-dimensional models of gut health. These models may elucidate, predict, or correct molecular and phenotypic interactions that were previously beyond the scope of single-omic or low-dimensional (e.g., 16S taxonomic) inquiries. Zeevi et al. demonstrated the value of the integrated data by training a machine learned (gradient boosting regression) model that combined blood biochemistry, dietary habits, anthropometrics, and physical activity, with the gut microbiome of 800 participants^35^. The resultant multi-dimensional model tested on an independent cohort (*n* = 100), predicted the personalized postprandial glycemic response to real-life dietary preference with ~2× higher correlation than the standard carbohydrate counting and meal caloric content models^35^. The significance of multi-omic interrogation through machine learning was further highlighted by Roelands et al., wherein high-confidence prediction of patient survivability (>97%) in colorectal cancer (CRC) was achieved by integrating microbiome signatures to the immunologic constant of rejection (or ICR i.e., the expression profile of a panel of 20 genes entailing T cell signaling, expression of chemokine ligands, cytotoxicity and counter-activation of immunoregulation)^36^. The role of *Ruminococcus* *bromii*, as a key microbiome signature for a favorable outcome in CRC was additionally highlighted. Furthermore, authors publicly released the study data as a “multi-omic cancer atlas“ spanning transcriptome, whole-exome, T cell receptor genes, whole genome sequencing and 16S amplicon gene profiles of tumor and matched healthy colon tissue^36^. Extending the application of multimodal data and AI modeling to accurate patient stratification, Ronen and colleagues developed a variational autoencoder-based deep learning (DL) method called multiomics autoencoder integration (maui) for stratifying cohorts into key CRC subtypes^37^. Authors anticipated the utility of such models in optimizing the design of clinical studies, especially toward personalized therapies for better treatment response. In another study, Wang et al. demonstrated the applicability of deep learning in identifying microbial variations, such as *Bacteroides coprocola* and *Clostridium* species, linked to the exposome, for detecting Pancreatic ductal adenocarcinoma (PDAC) via Multi-Omics Graph Convolutional Networks (MOGONET)^38^. MOGONET combined Graph Convolutional Network (GCN) and View Correlation Discovery Network, achieving high accuracy (0.82 ± 0.09), F1 score (0.84 ± 0.08), and AUROC (0.87 ± 0.09) in patient stratification for a cohort of 57 PDAC patients and 50 controls. MOGONET was reported to surpass traditional methods and microbiome-alone predictions^38^.

We opine that artificial intelligence and machine learning (AI/ML) will play a critical role in processing the vast multi-omics data coming from the ingestible microdevices. Incorporating the entire central dogma of gut microbiota through metagenomics, metatranscriptomics, metaproteomics, and metabolomics will provide a comprehensive view of microbial functions, community interactions, and host-microbe-drug cross talk. These insights may inform predictive models that could anticipate microbiome changes due to factors like diet and medication, guiding targeted therapy development. AI/ML algorithms may additionally pinpoint potential biomarkers tied to microbial patterns or diseases, enabling early detection, therapeutic strategies, and personalized treatment in gastrointestinal disorders. Finally, given the lack of simultaneously captured multi-omic datasets, the gut traversing miniature samplers may help enrich the existing omics data cataloging initiatives, like the Unified Human Gastrointestinal Genome^39^ and the Human Microbiome Project^40,41^ with the complete molecular snapshots of the human gut. Non-invasive gut sampling devices can elevate our understanding of the complex host-microbiome relationship to an entirely new level. We now summarize three critical areas of development that gut sampling devices would spur growth in and related frameworks required, as follows:

### Illumination of the microbial dark matter

Microbial dark matter, vis-à-vis the human gut, refers to the latent space of the human microbiome (including both taxa and genes or functions) that remains to be discovered or characterized^42^. The persistence of this gap may be attributed to multiple factors, notable being (i) the inability to culture and characterize all the GI microbes in metagenomic samples^29,42^ (ii) the inability to sample the diverse and challenging to access microniches of the gut^34^, and (iii) limited availability of databases for querying the total microbiome^30,43^. These limitations, as emphasized earlier, can be addressed by (i) microenvironment entrapment and culturomics aided by gut sampling microdevices (ii) gut-traversing ability of the miniature non-invasive pill-sized microdevices supported via peristalsis in difficult-to-reach GI niches and (iii) generation of the massive multi-omic datasets to reconstruct the complete picture of gut microbiome.

It is crucial to note that the gut microbiome is not functionally redundant^30^. The misconception regarding functional redundancy of the gut microbiota is essentially rooted in the current practice of mapping metagenomes to (incomplete) reference databases, restricting annotations to the common housekeeping and/or well-characterized genes. An ability to isolate spatially-resolved microbiome via ingestible microdevices, and then culturing it under laboratory conditions of entrapped microenvironments (or culturomics optimized media) shall accurately determine previously unknown specialist microbes as well as their functions (the dark matter). For instance, though the generalist SCFA production is conserved across many different microbiota species, oxalate and resistant-starch degradation are highly restricted to a handful of keystone species. Such specialist functions would remain unresolved for gut metagenomes in the absence of the identification of the associated keystone species like *Oxalobacter formigenes* and *Ruminococcus bromii*^30^. Ingestible gut sampling microdevices (and subsequent in-vitro functional characterizations) are perhaps well poised to establish the non-redundancy of the gut microbiome with high resolution.

### Precision in modeling systems biology of the gut

Computational models of gut ecology like BacArena, COMETS, MCM, studying the systems biology of the gut, simulate inter/intraspecies metabolic interactions, predict the trend of growth, nutrient assimilation, and anabolic/catabolic as well as the competitive fates of the members of the gut flora^44–46^. This modeling is based on the prior knowledge of biological networks, including genome-scale metabolic networks, cell signaling, gene co-expression networks etc^47^. Such frameworks, along with the in-silico agent-based models of the GI tract, like GutLogo^48^, act as virtual proxies of the GI tract, offer non-invasive, resource and time efficient approach for studying numerous hypotheses for any clinical study. Their development, however, requires multiple and benchmarked data streams of the GI microenvironment to mimic the complex ecosystem of the GI tract reliably. In this regard, the gut-sampling microdevices are expected to act as the real-time, reliable, and multimodal data-gathering sensors, which may provide the essential (precise) priors for modeling the metabolic fluxes, Bayesian processes, and even differential equations essential to these simulated models of the gut.

### Ethics and data security

Last but not least, given the highly personalised and multi-modal access to an individual’s molecular makeup, ensuring patient privacy, and responsible data management will be critical as the application of gut sampling microdevices advances. Training AI/ML models including the emergent information blackboxes — Large Language Models (LLMs) on publicly shared multi-omic databases poses a unique set of challenges. Monitoring intended usage and anonymising molecular identity of patients is just a tiny fraction of it. Collected data may additionally contain inherent biases including inter-individual variability in device actuation, apart from demographic imbalances, disparities in access to care, or historical inequities. Models derived from the biased data could further perpetuate incorrect recommendations, reinforcing health disparities. Increased transparency when reporting methodologies, targeted biomarkers, site of actuation, would not only help compare results from different studies but also aid data-integration strategies a Strengthen the Organization and Reporting of Microbiome Studies (STORMS) guidelines would be helpful in this regard^49^.

## Discussion

In summary, non-invasive gut sampling microdevices hold significant potential for transformative impacts on healthcare. Despite being currently underacknowledged, these devices can revolutionize healthcare by seamlessly integrating insights from microbiome research, predictive modeling, and personalized interventions. This integration unlocks novel therapeutic avenues, paving the way for precise, patient-centered treatments. The relevance of these microdevices becomes prominent particularly in the area of nutrition, auto-immunedisorders (mucosal immunology), and neurodegenerative diseases (gut-brain connection).

For instance, Dhakan et al. conducted a study highlighting the distinct composition of the Indian gut microbiome^50^. In their study of the North-Central Indian cohort, preferring a plant-based diet, associations with Prevotella were observed, along with enrichment of branched-chain amino acid (BCAA) and lipopolysaccharide biosynthesis pathways. Conversely, the Southern Indian cohort, following an omnivorous diet, showed associations with Bacteroides, Ruminococcus, and Faecalibacterium, with an enrichment in the short-chain fatty acid biosynthesis pathway and BCAA transporters. However, it’s important to note that the study used fecal signatures, which may not entirely represent site-specific microbiota composition. Additionally, the impact of comprehensive nutritional status, encompassing growth, nutritional blood biomarkers, dietary intakes, and feeding practices, on the gut microbiome in the Global South remains underreported in microbiome research.

In another study, for example, focusing only on the rectal microbiome of 10–18 month old children from urban slums of India, Huey et al. reported a high relative abundance of Proteobacteria, a phylum with potentially pathogenic species^51^. This dysbiosis was similar to that observed in preterm infants^51^. This suggests potential immaturity of the gut or a high inflammatory burden where the non-invasive sampling devices can probe upper and mid gut, bridging a clinical gap against protein energy malnutrition. Furthermore, given the higher-than-expected prevalence of diabetes and other metabolic non-communicable diseases (NCDs) in India, serious healthcare implications have been predicted that warrant urgent attention^52^. Regional healthcare policies and personalised interventions, such as GI sampling microdevices, could precisely map the (gut) biogeographical, regional and genetic diversity which cannot be achieved with stool sampling or focusing on colon alone. Overall, there is a pressing need to explore the comprehensive interplay between nutritional factors and the total gut microbiome in diverse populations, a gap that remains underreported in current microbiome research. This exploration becomes particularly crucial with the adoption of advanced technologies such as metabolic modelling, artificial intelligence and machine learning (AI/ML) to unravel intricate patterns and correlations within the vast multi-omic space of the gut microbiome.

**Fig. 4:**
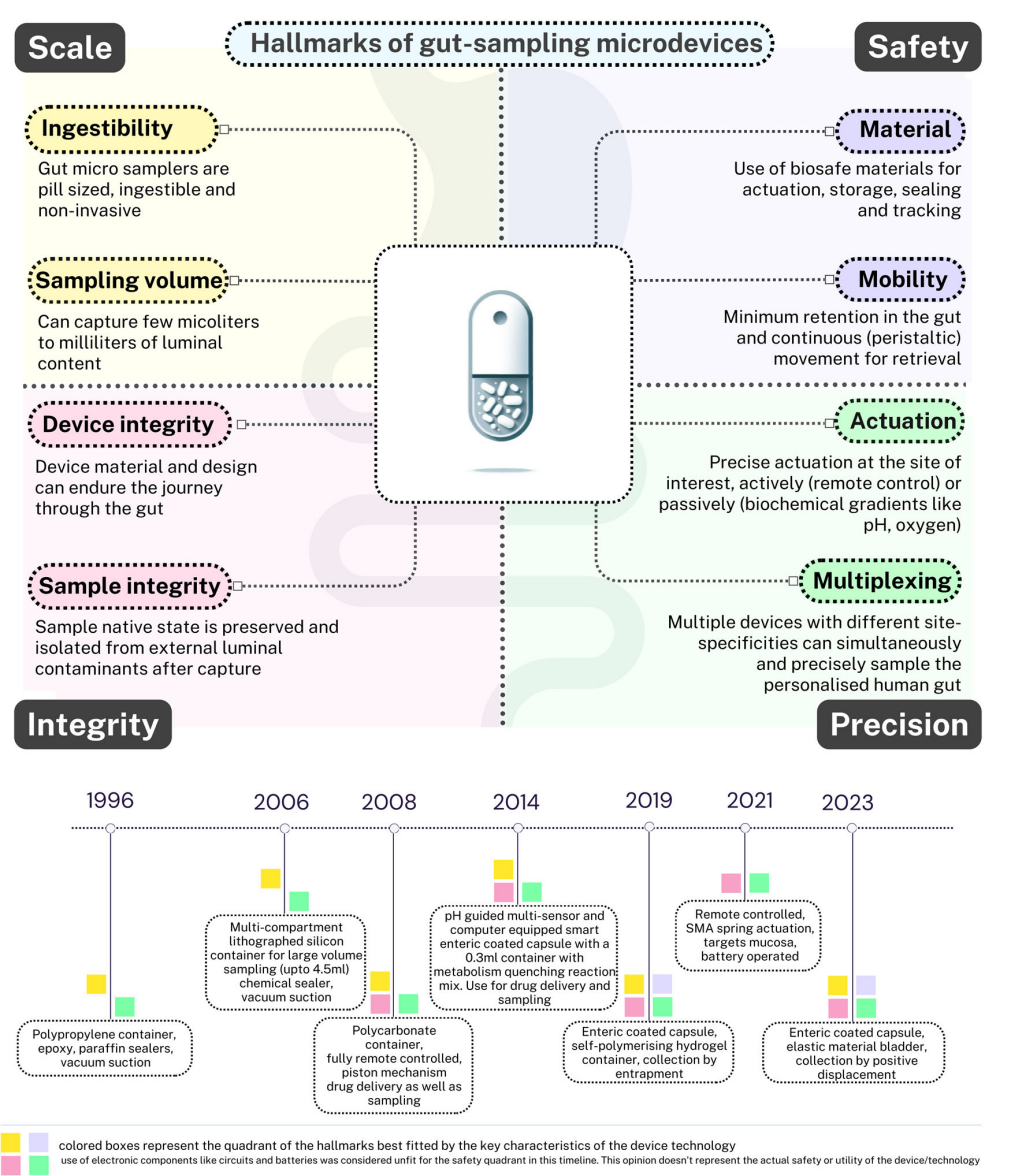
Hallmarks of gut-sampling microdevices. Top panel represents the four hallmarks of Scale, Safety, Precision and Integrity for developing in-situ samplers of human gut. Each hallmark is governed by two key traits underscoring a reliable development of the device. Bottom panel represents the timeline of the last three decades, highlighting the evolution of key technologies in the space of gut-sampling microdevices described in Table 1.

This perspective culminates in three recommendations: Enhancing transparency in omics methodologies for cross-study comparisons, addressing biases in AI/ML training, and promoting ethical frameworks for model training and patient-centric personal data. Gut sampling devices are well poised to emerge as vanguards of personalized treatment where clinicians, engineers, data scientists, and policy makers will have a key-role to play. We conclude by summarizing, in Fig. 4, key hallmarks that may guide the future developments in the space of in-situ GI tract sampling. Rest assured, the best is yet to come.
